# Ovarian Carcinosarcoma Mimicking Symptoms of Recurrent Diverticulitis: A Case Report

**DOI:** 10.7759/cureus.57948

**Published:** 2024-04-10

**Authors:** Riley Phyu, Harrison A Patrizio, Todd Schachter

**Affiliations:** 1 Department of Clinical Education and Assessment Center, Rowan-Virtua School of Osteopathic Medicine, Stratford, USA; 2 Department of Family Medicine, Rowan-Virtua School of Osteopathic Medicine, Stratford, USA

**Keywords:** unexplained abdominal pain, abdominal pain, diagnostic challenges, diverticulitis, malignant mixed müllerian tumor (mmmt), ovarian carcinosarcoma

## Abstract

Ovarian carcinosarcoma, also known as malignant mixed müllerian tumor, is a rare and highly aggressive form of ovarian cancer. This report discusses a case where initial misdiagnosis underscored the complexity of diagnosing this condition. The findings highlight the critical nature of considering ovarian malignancies in the differential diagnosis for postmenopausal women presenting with abdominal pain and altered bowel habits. The significance of utilizing advanced imaging techniques and tumor markers in the early detection of ovarian carcinosarcoma is emphasized, demonstrating how such strategies can substantially affect patient management and outcomes. This case also illustrates the effectiveness of a multidisciplinary approach in treating this challenging malignancy, contributing to our understanding and management of ovarian carcinosarcoma.

## Introduction

Ovarian cancer, the fifth leading cause of cancer-related deaths among women, continues to pose a significant health challenge [1-3]. In 2023 alone, an estimated 19,710 women in the United States were diagnosed with ovarian carcinosarcoma, with more than half presenting metastatic disease at diagnosis due to the often asymptomatic nature of the cancer in its early stages [2]. Within this spectrum of ovarian malignancies lies ovarian carcinosarcoma, also known as malignant mixed müllerian tumor (MMMT), a rare and aggressive subtype that accounts for 1-3% of cases [4,5]. Its tendency to manifest through nonspecific symptoms frequently leads to misdiagnosis, making the need for timely and accurate identification imperative for optimal patient outcomes [6]. In this case report, we present a unique case of ovarian carcinosarcoma that was initially mistaken for diverticulitis, a prevalent gastrointestinal condition, highlighting the diagnostic challenges inherent in this malignancy.

## Case presentation

A 67-year-old Caucasian female with a history of diverticulitis treated six years earlier and atherosclerosis visited the family medicine clinic, reporting persistent left lower quadrant abdominal pain for two weeks. The pain, considerably milder than her previous diverticulitis episode, was accompanied by occasional "shock" sensations. In response to her discomfort, she adjusted her diet to more neutral foods, hoping to alleviate the pain, but her symptoms have remained unchanged since their onset. She reported changes in bowel habits, including less frequent and smaller bowel movements that often required straining. However, she did not experience nausea, vomiting, diarrhea, fever, or shortness of breath.

During her initial visit, a physical examination revealed abdominal distension and tenderness in the left lower quadrant, with no signs of guarding or rebound tenderness. No imaging was ordered, assuming it was another bout of diverticulitis, so treatment with Augmentin for suspected recurrent diverticulitis was initiated, and a follow-up was recommended if no relief was found. She returned three days later, reporting no improvement. She was tolerating her Augmentin but reported feeling fatigued and experiencing rectal pressure. Her left lower quadrant was slightly tender to light palpation. She denied having a fever. A complete blood count (CBC) with differential and a CT scan of the abdomen and pelvis were ordered. CBC revealed a slightly low red blood cell count of 4.03 million per cubic millimeter (million/mm3), and a comprehensive metabolic panel returned normal results. A CT scan of the abdomen and pelvis disclosed a 10.1 x 9.4 x 8.7 cm heterogeneous solid mass in the right adnexa, likely of ovarian origin. The mass appeared to encroach on the sigmoid colon, with nodular infiltration of the omentum suggestive of metastatic involvement. There were no signs of inflammatory changes, bowel obstruction, or ascites.

The patient was referred to a gynecologic oncologist and reported symptoms reminiscent of a diverticulitis flare-up, increased abdominal girth, bloating, and left pelvic pain. A positron emission tomography/CT (PET/CT) scan from the skull base to the mid-thigh, a chest X-ray, and a cancer antigen 125 (CA 125) test were ordered. The chest X-ray showed no acute cardiopulmonary disease. The PET/CT scan revealed marked hypermetabolism within the large heterogeneous right adnexal mass, extending into the left adnexa, consistent with a malignant process. Incidental findings included left parapelvic renal cysts and diverticulosis, without signs of active diverticulitis. Small hypermetabolic lymph nodes were noted bilaterally in the axilla, likely reactive. The CA 125 test indicated an elevated level of 156 units per millimeter (U/mL).

A multidisciplinary approach was adopted, involving diagnostic laparoscopy and the potential for exploratory laparotomy, hysterectomy, bilateral salpingo-oophorectomy, and staging or cytoreduction based on intraoperative findings. The patient consented to the surgical plan. During surgery, a large mass was discovered in the right adnexa, adhered to the cul-de-sac, right pelvic side wall, and portions of the colon. A total abdominal hysterectomy, bilateral salpingo-oophorectomy, pelvic and para-aortic lymph node dissection, omentectomy, peritoneal biopsies, and optimal debulking were performed. Histopathological examination of the surgical specimens was crucial for diagnosis and treatment planning. The right adnexal mass fulfilled the criteria for carcinosarcoma, an MMMT, with immunohistochemistry confirming aberrant tumor protein 53 (p53) expression across both components. The staging, based on the International Federation of Gynecology and Obstetrics (FIGO) system, was determined to be IIIA2, indicating advanced disease but confined within the pelvic region. Subsequent staging, utilizing the FIGO system, classified the disease as stage IIIA2. This stage is characterized by the tumor's direct extension to the peritoneum outside the pelvis and/or metastasis to the retroperitoneal lymph nodes, indicating advanced disease but confined within the pelvic region. This classification underscores the importance of comprehensive surgical intervention and guides subsequent adjuvant therapy planning. The histopathological findings are depicted through a series of figures.

**Figure 1:**
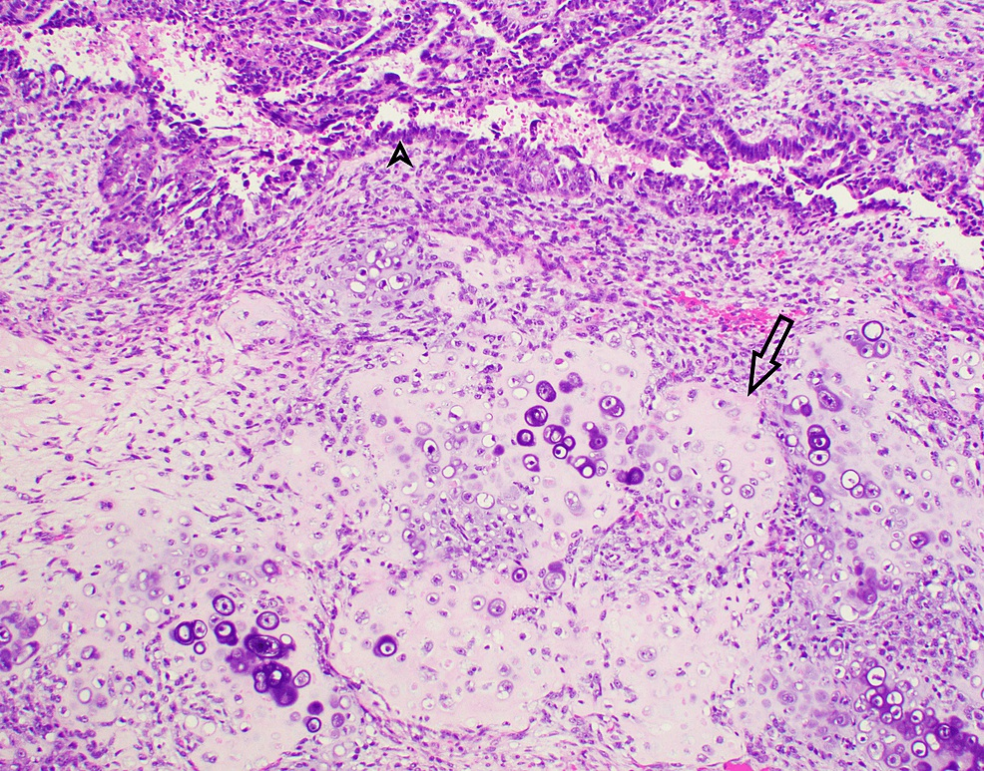
Carcinosarcoma-chondrosarcomatous component The carcinosarcoma of the ovary MMMT showed a carcinomatous/epithelial component (marked by an arrowhead) and a sarcomatous component in the form of heterologous malignant cartilage formation in this image (marked by an arrow). Both elements showed diffuse aberrant p53 staining by IHC stain (H&E and IHC stain, 200x). MMMT: malignant mixed müllerian tumor; p53: protein 53; H&E: hematoxylin and eosin; IHC: immunohistochemistry

Figure 1 illustrates chondrosarcomatous differentiation within the ovarian carcinosarcoma, highlighting diffuse aberrant p53 protein expression within the epithelial elements, corroborating the epithelial origin and sarcomatous differentiation of the malignancy.

**Figure 2:**
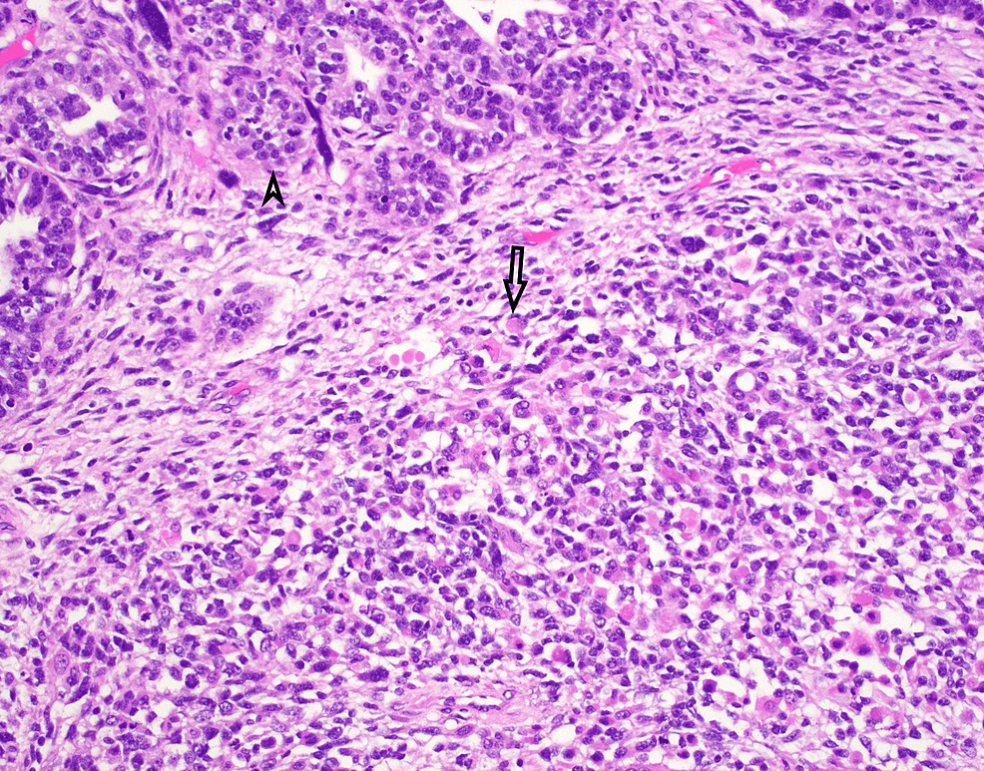
Carcinosarcoma rhabdo component The rhabodomyoblastic heterologous sarcomatous component present in this image (marked by an arrow) showed characteristic eosinophilic cytoplasm and was positive for desmin, myogenin, and MyoD1 by IHC. The arrowhead highlights the carcinomatous component (H&E and IHC stain, 200x). MyoD1: myogenic differentiation antigen 1; H&E: hematoxylin and eosin; IHC: immunohistochemistry

Figure 2 displays a subsection of the sarcomatous component with rhabdomyoblastic differentiation, evidenced by eosinophilic cytoplasm positive for desmin, myogenin, and myogenic differentiation antigen 1, adding to the histological heterogeneity.

**Figure 3:**
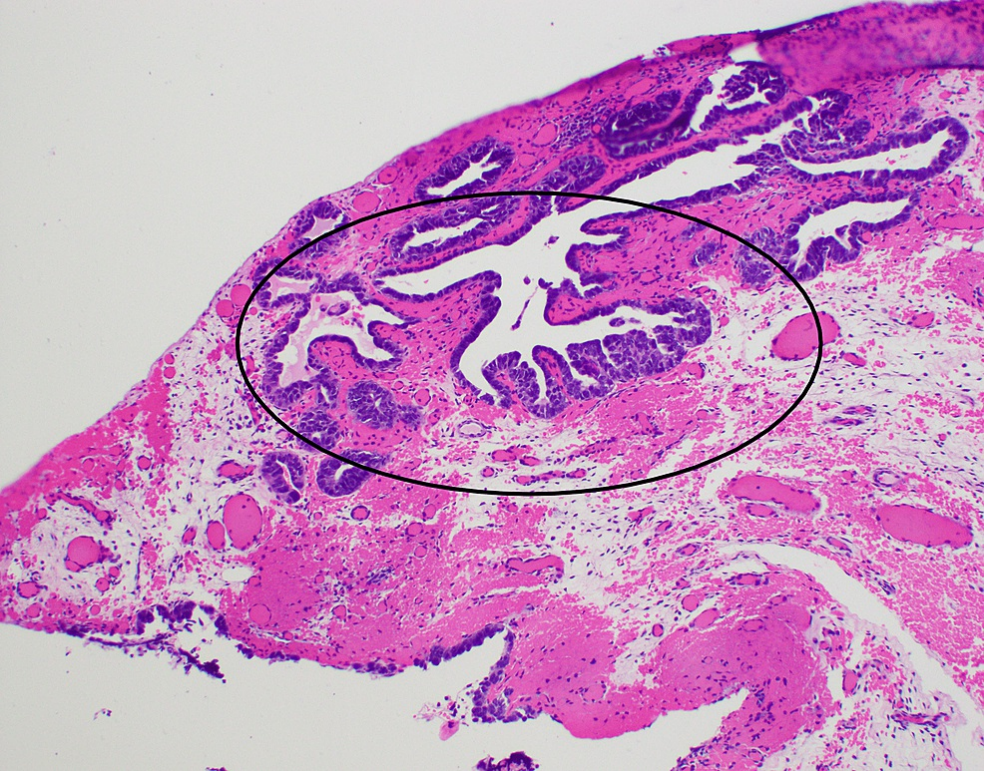
Fallopian tube STIC The right fallopian tube fimbriated end shows highly atypical cells lining the fallopian tube (circled). The cells were diffusely positive for p53, and the Ki-67 index was increased. The findings are consistent with the diagnosis of STIC (H&E stain, 200x). STIC: serous tubal intraepithelial carcinosarcoma; p53: protein 53; H&E: hematoxylin and eosin

Figure 3 identifies serous tubal intraepithelial carcinosarcoma (STIC) at the fimbriated end of the right fallopian tube, with cells exhibiting marked atypia and strong p53 positivity, suggesting a potential origin for the carcinosarcoma.

**Figure 4:**
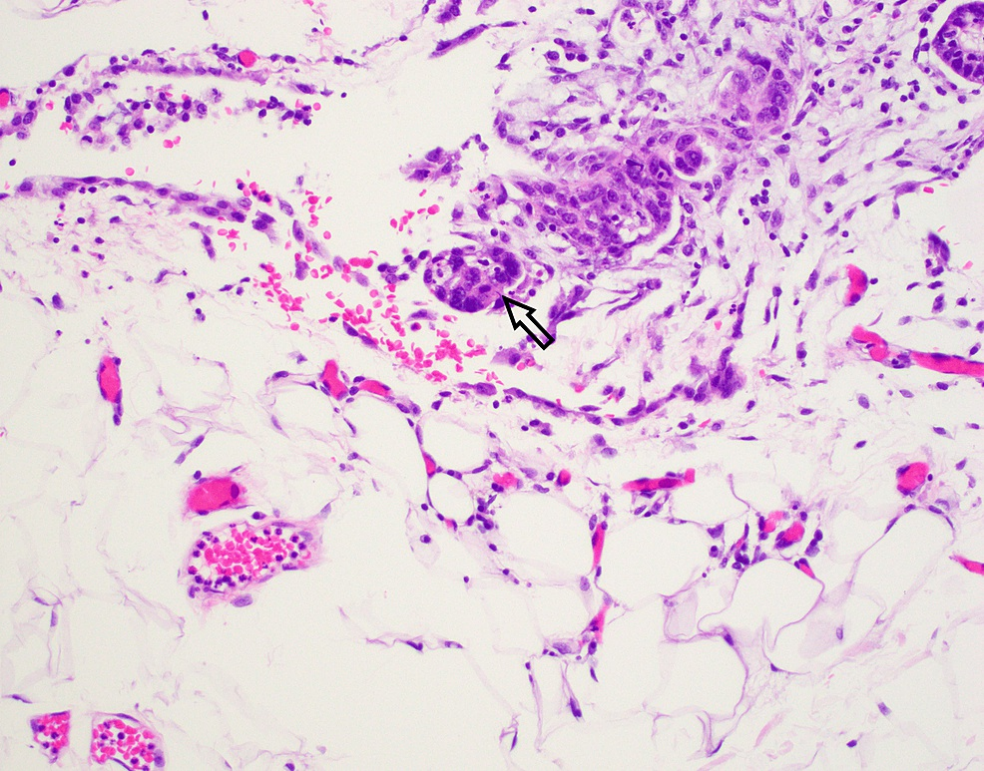
Omental involvement The carcinomatous component of carcinosarcoma (arrow) was seen involving the omental fibroadipose tissue (H&E stain, 200x). H&E: hematoxylin and eosin

Figure 4 captures the invasive behavior of the carcinosarcoma into the omentum, aligning with clinical and radiologic evidence of advanced disease and necessitating aggressive management.

These findings confirmed the diagnosis of ovarian carcinosarcoma with fallopian tube STIC and omental involvement, guiding the subsequent chemotherapeutic management plan. The patient's postoperative recovery was unremarkable, with no complications. Her abdominal pain significantly diminished within a day, and her recovery was marked by well-controlled pain, stable hemoglobin levels, and the ability to tolerate oral intake without complications. Discharged with appropriate instructions and anticoagulation therapy, she continued her recovery seamlessly at home. After an in-depth discussion regarding the next phase of treatment, she initiated her chemotherapy regimen, receiving the first of six planned cycles of taxol and carboplatin infusion chemotherapy approximately six weeks postoperatively. Despite experiencing and effectively managing an adverse reaction of constipation after the initial infusion, subsequent treatments were well-tolerated. Her most recent imaging results showed no progression of the disease, and the patient remains under close surveillance for six months while continuing her treatment journey. This case report has been reported in line with the Surgical Case Report 2020 criteria [7]. Written informed consent was obtained from the patient for publication of this case report and accompanying images.

## Discussion

Ovarian carcinosarcoma, or MMMT, represents a significant challenge in gynecologic oncology due to its aggressive behavior, rarity, and the complex nature of its diagnosis. This malignancy, accounting for only 1-3% of ovarian cancers, necessitates heightened clinical vigilance and a nuanced diagnostic and management approach beyond what is typically employed for more common ovarian cancers [4,5]. This case report highlights the diagnostic journey for ovarian carcinosarcoma, underscoring the importance of early consideration and comprehensive evaluation to mitigate the substantial diagnostic challenges this rare cancer presents [1].

The primary diagnostic challenge is the malignancy's non-specific symptomatology, often mirroring benign conditions and other gynecological malignancies with symptoms such as abdominal discomfort, bloating, and early satiety, leading to potential misdiagnoses [6]. This non-specific presentation emphasizes the need for clinicians to maintain a high index of suspicion, particularly in postmenopausal women presenting with unexplained abdominal or pelvic symptoms, to avoid delays in diagnosing this aggressive cancer [6].

Advanced imaging techniques and the evaluation of tumor markers, despite their lack of specificity, are critical in the diagnostic process, assisting in distinguishing ovarian carcinosarcoma from other potential conditions [7,8]. This approach is vital for facilitating earlier detection and initiating appropriate treatment, highlighting the indispensable role of a comprehensive diagnostic workup in managing this malignancy [7,8].

Upon diagnosis, the management of ovarian carcinosarcoma typically involves a combination of surgery and chemotherapy, mirroring the treatment strategies for other ovarian cancers. However, the prognosis for ovarian carcinosarcoma remains significantly poorer, underscoring the aggressive nature of this malignancy and the urgent need for effective management strategies [9,10]. This stark prognosis necessitates early and accurate diagnosis as a key factor influencing treatment outcomes, emphasizing the importance of exploring novel therapeutic options to improve patient outcomes [9,10].

A collaborative, multidisciplinary approach is paramount to optimizing patient care, ensuring that specialists across oncology, pathology, radiology, and gynecology contribute their expertise. This integrated approach not only enhances diagnostic accuracy and treatment efficacy but also provides comprehensive support to patients navigating the complexities of this formidable malignancy [1,5].

Ovarian carcinosarcoma demands proactive and discerning diagnostic strategies due to its non-specific presentation and the critical implications of diagnostic delays. This case report calls for ongoing clinician education and vigilance, leveraging advanced diagnostic tools, and fostering multidisciplinary collaboration to enhance early detection and management of ovarian carcinosarcoma. Further research into this malignancy's pathophysiology and targeted therapies is crucial for advancing care and improving the prognosis for patients with this challenging condition [4].

## Conclusions

The intricate case of ovarian carcinosarcoma presented here underscores the imperative need for healthcare professionals to exercise heightened vigilance and adopt an interdisciplinary strategy in their diagnostic processes. Given the malignancy's propensity for vague and non-specific symptomatology, which can lead to its misidentification as more common conditions such as diverticulitis, it is crucial that ovarian carcinosarcoma be considered in the differential diagnosis of patients presenting with ambiguous abdominal or pelvic symptoms. This approach is vital not only for the timely and accurate diagnosis of this aggressive cancer but also for initiating early and appropriate treatment strategies that can significantly impact patient outcomes. The insights derived from this case enrich our collective understanding of ovarian carcinosarcoma, highlighting the importance of early detection, the value of comprehensive evaluation, and the necessity for ongoing research to develop more effective therapeutic interventions. This case report reinforces the need for a collective and informed approach to navigate the complexities of diagnosing and treating ovarian carcinosarcoma, aiming to improve the prognosis and quality of life for affected individuals.
